# Using High-Resolution Future Climate Scenarios to Forecast *Bromus tectorum* Invasion in Rocky Mountain National Park

**DOI:** 10.1371/journal.pone.0117893

**Published:** 2015-02-19

**Authors:** Amanda M. West, Sunil Kumar, Tewodros Wakie, Cynthia S. Brown, Thomas J. Stohlgren, Melinda Laituri, Jim Bromberg

**Affiliations:** 1 Natural Resource Ecology Laboratory, Colorado State University, Fort Collins, CO, United States of America; 2 Department of Bioagricultural Sciences and Pest Management, Colorado State University, Fort Collins, CO, United States of America; 3 Graduate Degree Program in Ecology, Colorado State University, Fort Collins, CO, United States of America; 4 Department of Ecosystem Science and Sustainability, Colorado State University, Fort Collins, CO, United States of America; 5 Rocky Mountain National Park, Estes Park, CO, United States of America; University of New England, AUSTRALIA

## Abstract

National Parks are hallmarks of ecosystem preservation in the United States. The introduction of alien invasive plant species threatens protection of these areas. *Bromus tectorum* L. (commonly called downy brome or cheatgrass), which is found in Rocky Mountain National Park (hereafter, the Park), Colorado, USA, has been implicated in early spring competition with native grasses, decreased soil nitrogen, altered nutrient and hydrologic regimes, and increased fire intensity. We estimated the potential distribution of *B. tectorum* in the Park based on occurrence records (n = 211), current and future climate, and distance to roads and trails. An ensemble of six future climate scenarios indicated the habitable area of *B. tectorum* may increase from approximately 5.5% currently to 20.4% of the Park by the year 2050. Using ordination methods we evaluated the climatic space occupied by *B. tectorum* in the Park and how this space may shift given future climate change. Modeling climate change at a small extent (1,076 km2) and at a fine spatial resolution (90 m) is a novel approach in species distribution modeling, and may provide inference for microclimates not captured in coarse-scale models. Maps from our models serve as high-resolution hypotheses that can be improved over time by land managers to set priorities for surveys and removal of invasive species such as *B. tectorum*.

## Introduction

*Bromus tectorum* L. is an invasive winter annual grass that was introduced into the United States from Eurasia in the late 19^th^ century. While the current distribution of *B*. *tectorum* extends throughout the United States, it is particularly abundant in the Intermountain West [1], and it is listed as a Class C noxious weed in the state of Colorado [2]. Prior research has linked *B*. *tectorum* abundance and distribution to climate [3,4], roads and trails [5,6], elevation [5,6], vegetation community type [6], and soils when moisture is limiting [3,7].

The winter annual life history of *B*. *tectorum* is not common amongst grass species in the Intermountain West. It may complete its life cycle as a winter or spring annual depending on temperature and water availability [1,2]. Autumn germinating individuals overwinter as semi-dormant seedlings; therefore these plants may begin growing earlier in the spring than native warm-season grass seedlings. Plant traits that harbor an advantage in novel climates include (1) high growth rate, (2) wide climatic or environmental tolerance, (3) short generation time, (4) prolific or consistent reproduction, (5) modified seed size, (6) effective dispersal, (7) uniparental reproduction capacity, (8) no specialized germination requirements, (9) high competitive ability, and (10) effective defenses against natural enemies [8]. Seven of these traits are exemplified by *Bromus tectorum* (it has an after-ripening requirement that stimulates germination; [1]). Furthermore, evidence from studies across a broad range of ecosystems suggests a significant shift upward (in elevation) and poleward (in latitude) for some plant species distribution in response to climate change [9,10], including *B*. *tectorum*, which has been documented recently at increasing elevations [5,6]. Rapid climate change may facilitate invasive species such as *B*. *tectorum* with high phenotypic plasticity and rapid growth from seedling to sexual maturity, increasing its competitive advantage over native species [1].

Species distribution models (SDMs) may enhance our understanding of the environmental variables that most influence the bioclimatic niche of a species, a subset of its fundamental ecological niche defined by associations between aspects of climate and known species occurrences [11]. The fundamental ecological niche of a species is defined as the multivariate space occupied by the species, including the environmental conditions associated with population maintenance [12]. Through the inclusion of future climate variables in SDMs, we may visualize the potential future geographic distribution of a species [13].

The Maximum entropy model, or MaxEnt [14], is a machine learning SDM that determines the probability distribution of a species over geographic space by estimating this distribution under the maximum entropy principle (i.e., exponential distribution given linear combination of features; equivalent to the Gibbs distribution). This SDM is of particular value when examining an introduced species such as *B*. *tectorum* because it requires only species presence data, whereas other SDMs such as logistic regression require presence and absence data. Models that include absence locations do not necessarily indicate unsuitable habitat, rather that the species has not had time or resources for broad dispersal [15].

There are many reasons that MaxEnt was chosen for our study. MaxEnt is a non-parametric model and can automatically include interactions among both continuous (e.g., climate) and categorical (e.g., vegetation community type) variables [16], is effective with small sample sizes [17–20], and has been shown to outperform or at least preform as well as many other modeling algorithms for SDMs of terrestrial plants, birds, bats, reptiles, and diseases [21–24]. In addition, inclusion of static and dynamic variables other than climate, such as vegetation community type and distance to roads may enhance the predictive capabilities of SDMs [25,26]. Similar to other species distribution models, MaxEnt can also be applied to future climate modeling [23,27]. Process-based mechanistic niche models such as CLIMEX also provide powerful tools for evaluating the potential effects of climate change on species distributions [28–30]; however these SDMs may not be ideal for use in a National Park. Correlative models such as MaxEnt can be fit to existing occurrence data whereas process-based models typically require detailed experimental data that may not be available for an introduced species in a National Park [31]. Furthermore, the finest resolution of the climate surfaces in CLIMEX are 1km[32], which may not be appropriate for modeling climate effects on species distribution in National Parks.

When managing for invasive species at the landscape scale, regional weather patterns important to fecundity may not be captured in coarse-resolution data [33–36]. Capturing climate refugia for species in areas with widely varying elevation such as Rocky Mountain National Park may be improved by using fine-scale data [10,37–39]. ClimateWNA software provides climate data for point locations, time series, and climate surfaces for Western North America [40]. This software was developed using downscaling algorithms (delta approach; for methods see [40]) where baseline climate data (PRISM and ANUSPLIN grids), historical data (CRU TS 2.1), and future projected data (Coupled Model Intercomparison Project; IPCC 2007) are interpolated as the difference from a common reference period, with their accuracy tested against local weather station data. Furthermore, partial derivative functions of temperature change along elevation gradients are incorporated in these methods, making them ideal for areas such as Rocky Mountain National Park [40].

Species distribution models are powerful tools in evaluating the bioclimatic niche of a species; however the assumption of niche conservatism should always be considered when projecting these models into future potential environmental space [41]. To address this concern, ordination methods may be used to estimate the maximum variance of climatic predictors and climatic niche overlap between current and future potential distributions [42]. Thus, we may examine the ordinal climatic niche space of a species in a given area currently, overlay it with the ordinal climatic niche space of the same species given the future climate projections, and evaluate the overlap and shift of this niche space. These niche comparisons may elucidate the potential distributional progression of a species and are useful tools to augment SDMs.

Based on preliminary analysis of current *B*. *tectorum* distribution in Rocky Mountain National Park (hereafter, the Park) and projected climate change in the Park, the primary hypothesis driving this study was that this alien invasive grass may continue to be problematic in current disturbed areas of the Park well into the future, and expand its bioclimatic niche as the climate changes in the Park. Our objectives were to: (1) evaluate the current bioclimatic niche including climatic variables that have a significant influence on *B*. *tectorum* occurrence in the Park using MaxEnt and high-resolution climatic data generated from ClimateWNA, (2) model the potential bioclimatic niche of *B*. *tectorum* in the Park for the year 2050 based on climate change, and (3) create a high-resolution map of *B*. *tectorum* habitat in the Park both now and in the future for use by Park managers. We also sought to better understand the usefulness of ordination methods to evaluate niche dynamics in ordinal space.

## Methods

### Study Area

Rocky Mountain National Park covers about 1,076 km^2^ in northern Colorado, USA, at approximate latitudes 40°10’N to 40°32’N. The elevation varies greatly in the Park, from 2,300 m to 4,345 m at the summit of the highest peak (Longs Peak) and the Continental divide separates the Park with 60% on the east slope and 40% on the west slope. At Estes Park climate station (station #052759 at 2364 m elevation; [43]), mean annual temperature from 1981–2010 was of 6.9°C, and mean annual precipitation was 41.8 cm. Montane, subalpine, and alpine ecosystems blanket the Park, yielding habitat to a wide variety of flora. Rocky Mountain National Park lists 28 alien plant species as common to abundant including *B*. *tectorum* [44]. The United States National Park Service defines an alien species as “those that occur in a given place as a result of direct or indirect, deliberate, or accidental actions by humans” [44]. We received a scientific research and collecting permit from Rocky Mountain National Park to proceed with this study (ROMO-2013-SCI-0038).

### Species Occurrence Data

Occurrence data for *B*. *tectorum* were combined from four prior field surveys in the Park conducted in 1996, 1999, 2007, and 2008 (n = 211; [6]). These surveys were conducted using a modified-Whittaker plot design [45]. We assumed these data were from populations that have not currently filled the entire bioclimatic niche due to dispersal limitations [46]. Additional *B*. *tectorum* occurrence data within a 100,000 m perimeter of Park boundaries were evaluated for inclusion in the study; however it was concluded that no new ecosystem types would be represented by these locations.

### Environmental Variables

**Fig 1:**
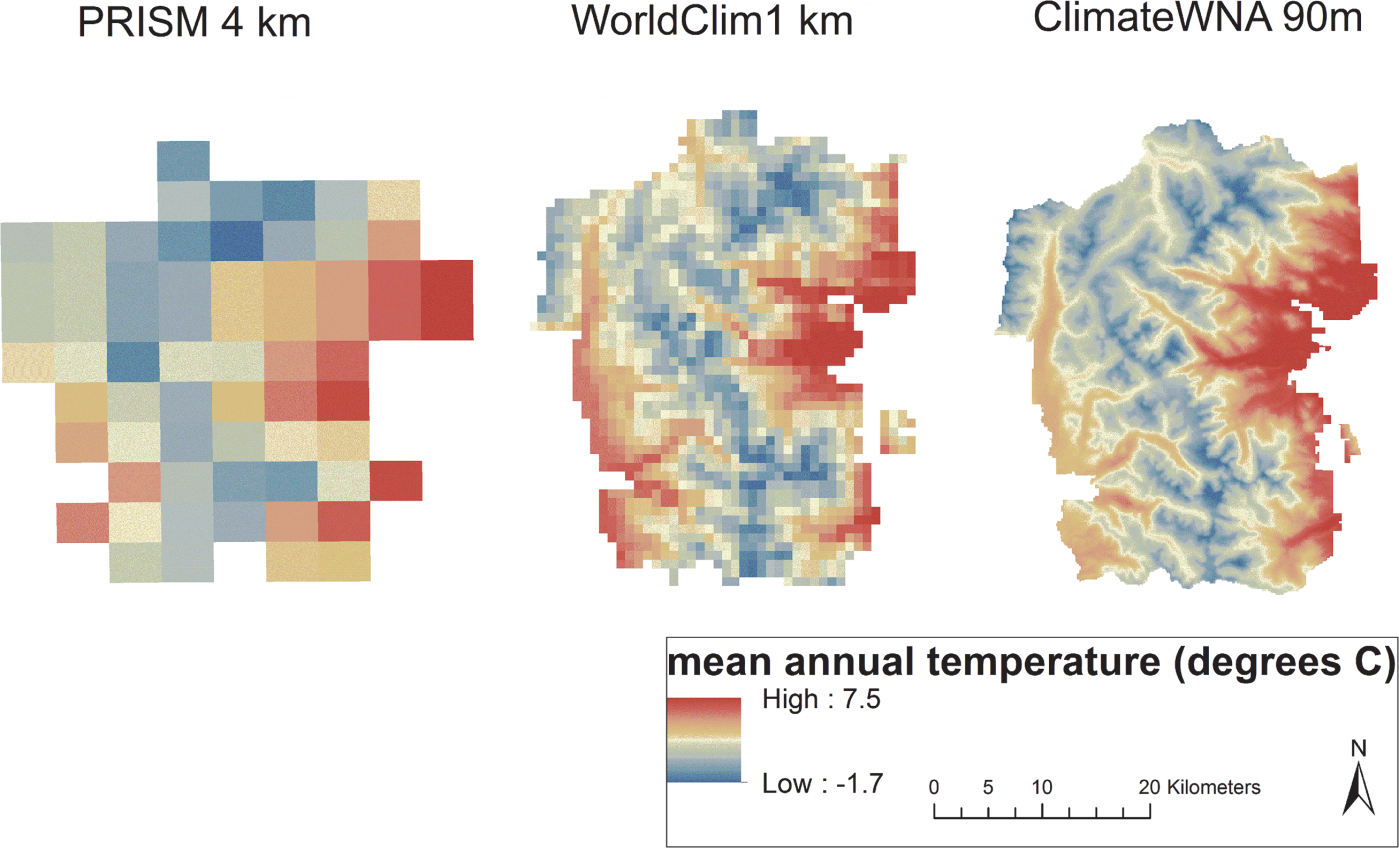
Comparison of mean annual temperature (MAT) at three spatial resolutions (4 km, 1 km, and 90 m) for climate normals 1981–2010 in Rocky Mountain National Park, Colorado. Data Sources: PRISM Climate Group (http://www.prism.oregonstate.edu/) WorldClim (http://www.worldclim.org/) and ClimateWNA (http://climatewna.com/).

We used current data (climate normals 1981–2010) encompassing 21 annual and 48 seasonal bioclimatic (continuous) variables at a 90 m resolution generated using ClimateWNA v. 4.72 program [40], distance to roads and trails (continuous), and vegetation community type (categorical) variables (S1 Table). Distance to roads and trails was included as a surrogate for *B*. *tectorum* propagule pressure because the seeds of *B*. *tectorum* readily attach to vehicle tires, hiking boots, and animal fur [5,6]. The 90-m spatial resolution was chosen for this study after an evaluation of climate data at a 4 km [47]and 1 km [48] determined finer climate refugia for the Park may not be captured at these spatial scales (Fig. 1). The 90-m spatial resolution resulted in 133,849 (90 x 90 m) grid cells, each with a unique value for the 71 variables. Elevation data [49], a proxy for climate, was not included in model development, but was used to extract all data from ClimateWNA, and evaluated post-modeling to gain insight concerning the potential elevational limits of *B*. *tectorum* in the Park.

Future climate scenarios (year 2050) from six global circulation models (GCMs) were extracted at a 90 m resolution from ClimateWNA. These included two runs of CCCMA (Canadian Centre for Climate Modeling and Analysis), MIROC-H (Centre for Climate Research, Japan), MRI (Meteorological Research Institute, Japan), BCCR (Bjerknes Centre for Climate Research, and GFDL (United States National and Atmospheric Administration Geophysical Fluid Dynamics Laboratory). Global circulation models incorporate energy flux measurements between the sun, atmosphere, and earth’s surface in algorithms that compute surface conditions. For all GCMs used in this model, the A2 climate change scenario was selected as this scenario follows observed trends in atmospheric carbon dioxide concentrations in the mid-2000s [50]. All variables considered were projected in Universal Transverse Mercator (UTM) coordinates in NAD83 datum (to match the *B*. *tectorum* occurrence data) using geographic information system format (ArcGIS v.10; ESRI, Redlands, CA, USA). Although there is uncertainty associated with any future climate scenario, these data provide the most reasonable predictions given our current understanding of future conditions.

### Statistical Analysis and Spatial Modeling

We used R v.3.0.1 statistical software (R Core Team 2012) to calculate Pearson correlations (Hmisc package; Frank Harrell) among the 69 climatic variables. All correlation values (r) were statistically significant (p < 0.001). When two or more variables were highly correlated (|r| ≥ 0.70), variables with the lower biologic relevance to *B*. *tectorum* was dropped [51]. Highly correlated variables do not add new information to SDMs, and their exclusion is the first step in determining the most parsimonius model. The distance to roads and trails and vegetation community type variables were not included in the Pearson correlation analysis (the former was retained to represent propagule pressure; the latter was a categorical variable). The correlation analysis resulted in nine variables for inclusion in the initial SDM: mean annual temperature, continentality (temperature difference between mean warmest month temperature and mean coldest month temperature,°C), summer chilling degree-days, beginning of frost-free period, mean summer precipitation, spring heating degree days, winter reference evaporation, distance to roads, and vegetation community type.

These nine variables were included in MaxEnt along with the *B*. *tectorum* occurrence data. An initial SDM was run in MaxEnt (one run; raw output setting) to acquire lambda values used in ENMTools v.1.3 [52] to calculate Akaike’s Information Criterion (AICc; [53]) for a model fit with all nine variables, eight, seven, six, five, four, three, two, and one of the variables, respectively (S2 Table). This method selects the fitted approximating model that is estimated to be closest to the unknown truth on average (i.e. the most parsimonious model). The model that was most parsimonious in our case (lowest AICc value) had six variables: mean annual temperature, continentality, beginning of frost-free period, mean summer precipitation, spring heating degree days, and distance to roads and trails.

These six variables were then incorporated into MaxEnt along with the *B*. *tectorum* occurrence data. The same six variables were included as projection layers for the year 2050 for all six GCMs. MaxEnt requires the user to specify a background for the study area from which the algorithm will select random points that are assumed as ‘pseudo-absences’. We set MaxEnt to select 10,000 random background points from the entire Park. The *B*. *tectorum* occurrences used in this study were collected using stratified random sampling in the Park, and personal communication with Park staff verified that these invaded areas were the only ones reported for the Park at the time these occurences were sampled, justifying this background point selection method. MaxEnt allows the user to change default settings based on study objectives [16,54]. We changed the following settings: (1) created response curves to evaluate *B*. *tectorum* response to individual variables, (2) conducted jackknife procedure to measure variable importance, (3) selected a random seed, (4) set random test percentage at 10 to evaluate model performance and reduce bias (90% of the data trained the model), (5) set replicates at 100 so that model results would not be dependent on a single sample and to ensure variability, (6) replicated run-type was set as subsample, (7) chose that plot data be written, (8) set maximum iterations to 5,000 allowing the model adequate time for convergence (prevents over- or under- prediction of correlations), (9) selected that background predictions be written, and (10) conducted ‘fade by clamping’ to ensure consistency in probabilities for the future climate projections.

MaxEnt produced seven continuous surface ASCII files; one for current probabilities of occurrence and one for each year 2050 GCM (i.e. six future climate scenarios). These ASCIIs contained relative probabilities of *B*. *tectorum* presence predicted for each 90-m pixel of the study area. Using ArcGIS v.10, the seven ASCII files were converted to binary maps in raster format, where 1 = suitable habitat and 0 = unsuitable habitat. This classification was based on the 10^th^ percentile training presence logistic threshold (= 0.32) produced by the MaxEnt model. Finally, the six year 2050 GCM rasters were combined and reclassified into non-habitat, decreasing, increasing, and stable habitat for *B*. *tectorum* in the Park using an ensemble approach [55].

### Model evaluation

To evaluate the final MaxEnt model with six variables compared to random expectations we calculated a partial AUC (area under receiver operating characteristic curve) ratio (pAUC) following [56]. A pAUC value of 1 indicates the model preformed no better than random; values >1 indicate the model preformed better than random. Partial AUC considers only the portion of the AUC curve that corresponds to model predictions rather than commission error rate (i.e. ratio of incorrectly predicted absence data to all absence data), which is not applicable for presence-only SDMs. For the pAUC analysis, 80% of the *B*. *tectorum* occurrence data (n = 169) were randomly selected to train a MaxEnt model using 10-fold cross validation. The remaining 20% of the data (n = 42) were used to test the logistic predictions of this model. The modeled suitability values of each testing point are used to calculate pAUC, and we ran 1000 iterations with 50% of the points resampled with replacement for each bootstrap.

To prepare data for a principle components analysis (PCA), 10,000 random points were selected from the entire study area of the Park, and current and future (year 2050; ensemble from averaging six GCMs) values for the five non-correlated climatic variables were extracted for these points in ArcMap v.10. Using R v.3.0.1, a PCA analysis was conducted for these variables to reduce dimensionality; multivariate attributes were reduced to two values (each variable was regarded as constituting a different dimension, in a p-dimensional hyperspace). The *niche*.*overlap*, *occ*.*prep*, and *niche*.*dynamic* functions in R were utilized and code was modified from [42]. This method converted the *B*. *tectorum* presence points (n = 211) to density values via kernel smoothing. The density values were then ordered along PCA axes of the current environmental grid (10,000 random background points) and then the future (2050) environmental grid (same 10,000 random background points). The current and 2050 models were overlaid to determine the extent of *B*. *tectorum* niche overlap in ordinal space, between current and future climates. This PCA analysis does not substantiate the results of the Maxent model; rather it provides another tool to evaluate the potential future niche of a species in ordinal space (i.e., the fundamental niche) rather than geographic space.

## Results

### Variable correlation and parsimony analysis

Out of 69 climatic variables, seven were found to be uncorrelated using Pearson correlation (|r| ≤ 0.70): mean annual temperature, continentality (temperature difference between mean warmest month temperature and mean coldest month temperature,°C), summer chilling degree-days, beginning of frost-free period, mean summer precipitation, spring heating degree days, and winter reference evaporation. The AICc analysis indicated a model with five of these seven climatic variables (mean annual temperature, continentality, beginning of frost-free period, mean summer precipitation and spring heating degree days) and the distance to roads and trails variable would be most parsimonious. Thus, these six variables were used in the final MaxEnt model.

### Final MaxEnt model with six variables

**Fig 2:**
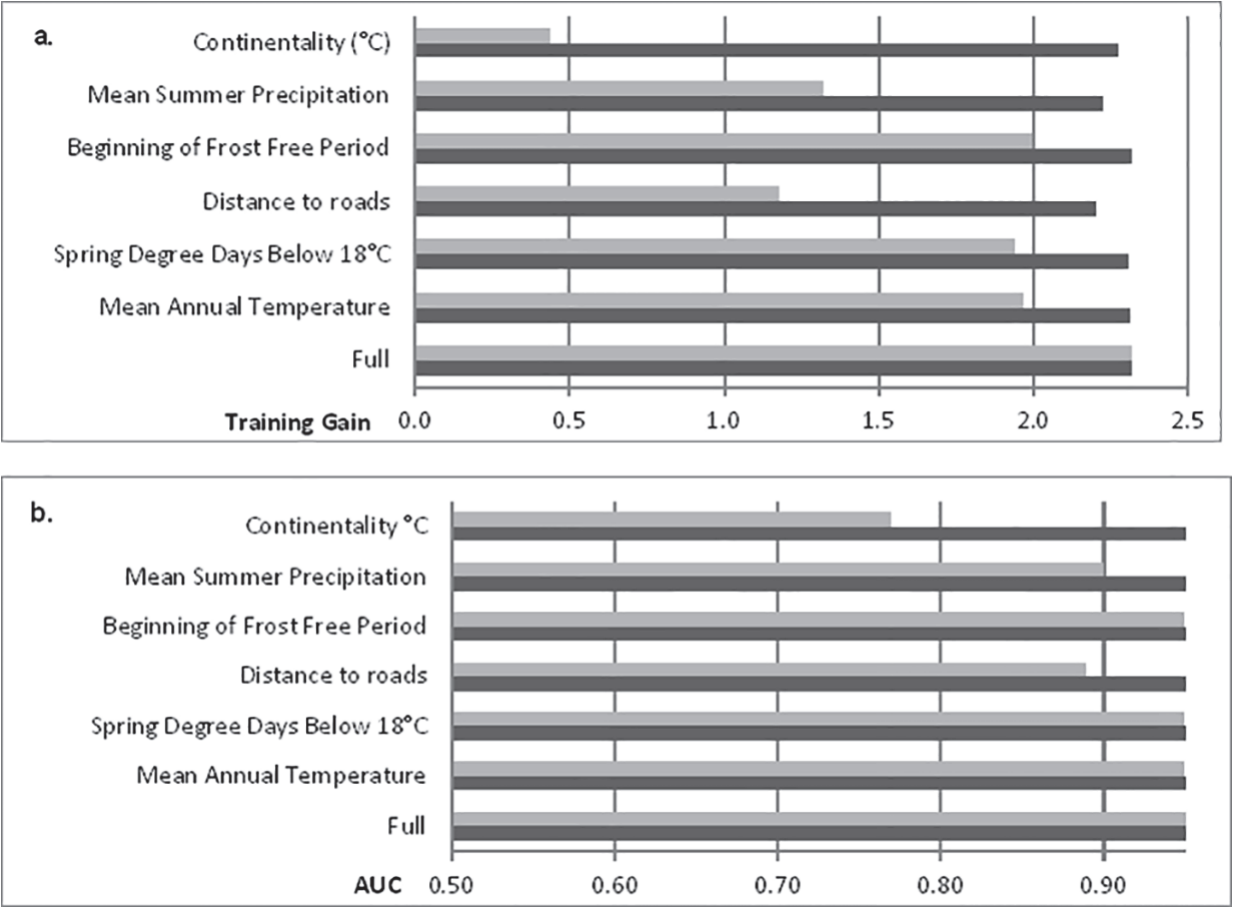
Variable contribution to training gain (a) and area under curve (b). Gray bars indicate how well the model performs with only that variable, versus a full model. Values shown are averaged over 100 replicate MaxEnt model runs.

The average pAUC value for the best model was 1.77 (± 0.0005), indicating the MaxEnt algorithm performed exceptionally well in discriminating areas as suitable *B*. *tectorum* habitat. Mean annual temperature had the greatest contribution to the model (43.7%), followed by spring degree days below 18°C (19.5%; Table 1). Distance to roads and beginning of frost-free period contributed equally to the model (13.5%), and mean summer precipitation contributed 6.3%. Continentality only contributed 3.5% to the model; however we felt confident in its inclusion because of the low AIC value associated with the six variable model compared to a model with more or fewer variables. Model response to the top predictor variable (i.e. mean annual temperature) indicated the probability of *B*. *tectorum* presence in a given cell is greater than 50% when mean annual temperature is between 5.5 and 7.5°C. Likewise, spring degree days below 18°C between 1000 and 1200 yielded at least 50% logistic probability, as well as beginning of frost-free period Julian date between 145 and 165, and distance to roads less than 1000 m. Mean summer (May—Sept.) precipitation and continentality showed the most conservative thresholds for *B*. *tectorum* response. Mean summer precipitation greater than 250 mm and less than 300 and continentality greater than 21°C but less than 22°C yielded greater than 50% logistic probability, and while their significance in the model was lower than the other four variables these narrow thresholds merit further investigation. Jackknife output confirmed the importance of mean annual temperature, spring degree days below 18°C, and beginning of frost free period to the final model (i.e., higher training gain and test AUC value; Fig. 2).

**Table 1:** Variables and their relative contribution in the final MaxEnt model.

Variable	Percent Contribution^1^	Permutation Importance
Mean annual temperature	43.7	17.2
Spring degree days below 18°C	19.5	5.1
Distance to roads	13.5	34.1
Beginning of frost-free period	13.5	6.4
Mean summer precipitation	6.3	23.7
Continentality	3.5	13.4

^1^ percent contribution is calculated as the increase in regularized gain added to the contribution of the corresponding variable for each of the 5000 iterations of the model (subtracted if the change to the absolute value of lambda is negative).

### Current and future potential suitable habitat of B. tectorum in the Park

**Fig 3:**
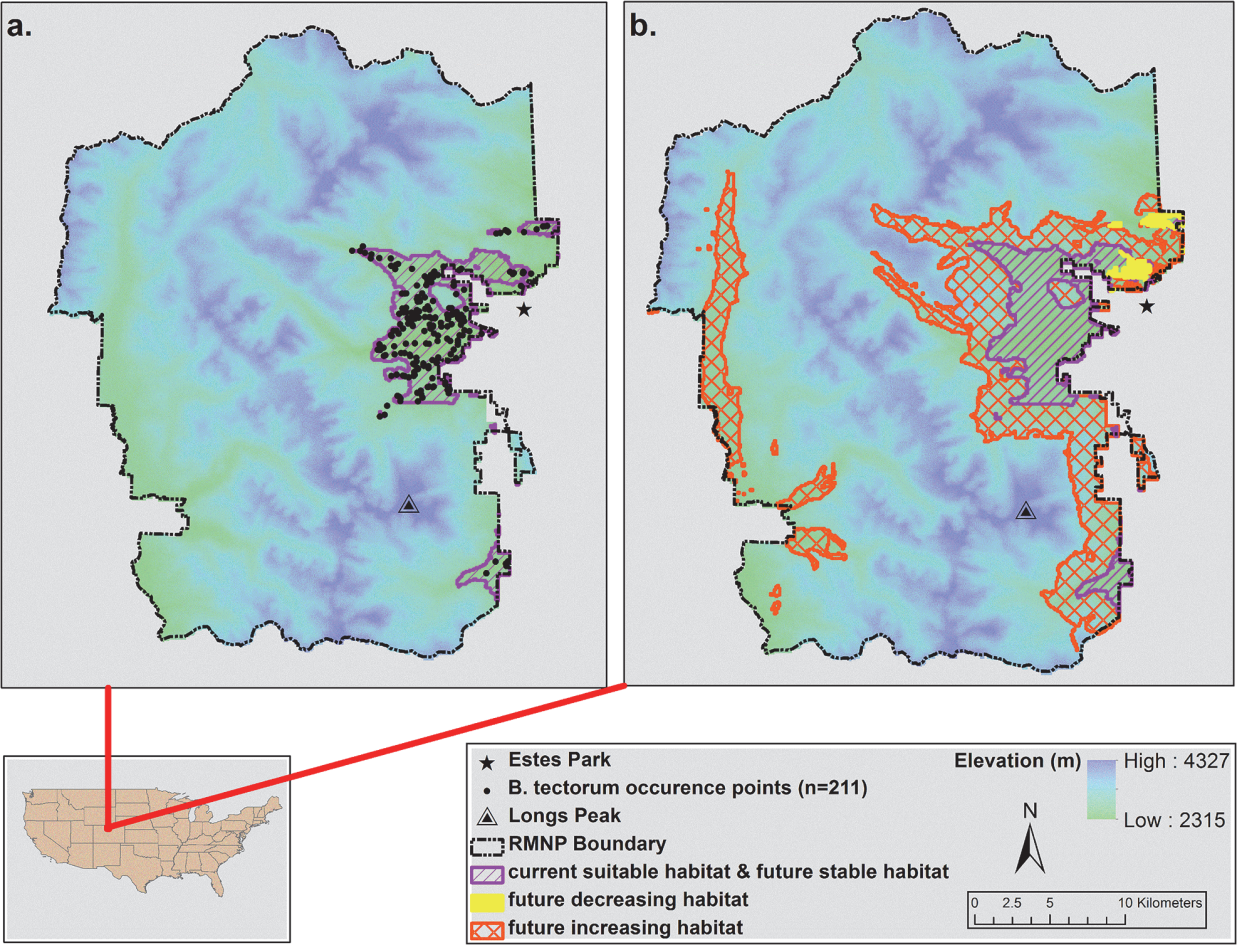
*Bromus tectorum* current suitable habitat in Rocky Mountain National Park (a) and increasing, decreasing, stable, and unsuitable habitat for the year 2050 (b) based on MaxEnt model outputs. Elevation has been included for reference (coordinate system NAD 1983 UTM Zone 13 N).

The current suitable habitat for *B*. *tectorum* in the Park is 62.2 km^2^, and in the future (year 2050) our ensemble model indicated 219.4 km^2^ of the Park as suitable habitat (Fig. 3). In the future model, 5.5% of the Park currently suitable remains stable habitat for *B*. *tectorum* (59.1 km^2^) while 0.3% decreases in suitability (3.1 km^2^). An additional 14.9% of the Park becomes suitable habitat (160.3 km^2^) compared to current conditions. Although elevation (a proxy for climate) was not included in the model, we overlaid the final model results on a digital elevation model of the Park to evaluate the potential elevational limits of *B*. *tectorum* given future climates. Currently, *B*. *tectorum* has been found up to 2,800 m elevation in the Park, and the current suitable habitat MaxEnt results agree with this. The future ensemble model indicates *B*. *tectorum* may reach nearly 3,300 m in elevation by the year 2050.

### Principle components analysis

**Fig 4:**
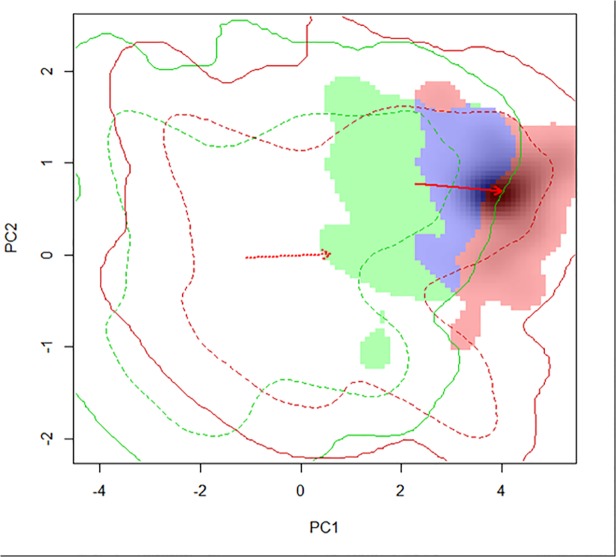
Principle component analysis (PCA) of niche overlap (blue) for *B*. *tectorum* in Rocky Mountain National Park based on current (green) and future (red) climate space. Averages for five climatic variables from six global circulation models (GCMs) were included in this analysis: mean annual temperature, spring degree days below 18°C, beginning of frost-free period, mean summer (May-Sept.) precipitation, and continentality (see methods for description of GCMs).

Relative occupancy for the current realized and year 2050 projected realized niches of *B*. *tectorum* in the Park are shown along each axis of the PCA model (Fig. 4). The PCA indicated both niche conservation (D = 0.22 or 22% overlap between current climate niche space and future ordinal niche space) and a shifting niche for *B*. *tectorum* in the Park between current and future conditions. Thus, 22% of the ordinal climatic niche of this species may remain in the Park under future climates, and 78% of this niche space may shift.

## Discussion

In this study we used MaxEnt model to integrate species occurrences and high-resoluton (i.e. 90 m) current and future climatic and other environmental data layers to develop current and future potential habitat distribution maps for *B*. *tectorum*. Compared to current conditions, in the year 2050 an additional 14.9% of the Park will be suitable habitat for *B*. *tectorum* (160.3 km^2^). Interestingly, the final MaxEnt model indicated some areas of the Park that are currently sutiable may no longer be suitable *B*. *tectorum* habitat in the future. Therefore, it is likely one of the most significant climatic variables may surpass an upper or lower value threshold for *B*. *tectorum* survival given its probability of presence. For example, the strongest predictor in the MaxEnt model, mean annual temperature may exceed the upper threshold of 7.5°C under future potential conditions in some areas of the Park. The distribution of both C3 and C4 grasses has been explained in previous studies primarily by mean annual temperature [57,58]. *Bromus tectorum* is a C3 grass, a group whose relative abundance tends to decrease with increasing mean annual temperature [58]. The raw ClimateWNA data indicated that mean annual temperature will increase for most of the Park in all six GCMs. Our model forecasts suitable habitat for *B*. *tectorum* at increasing elevations in the Park for the year 2050, suggesting this alien grass may respond to increasing mean annual temperatures by moving upward in elevation to maintain its niche space. This model showed suitable habitat for *B*. *tectorum* up to 3,300 m elevation, indicating this invasive grass may become problematic in subalpine ecosystems of the Park. *Bromus tectorum* has already been found at 3,000 m elevation in the Himalyan mountains [59].

The significance of all six variables that were selected through our parsimony analysis to *B*. *tectorum* ecology indicates the importance of careful scrutiny and correlation analysis of available environmental data for a study site of interest *a priori*, as other authors have indicated [60,61]. In particular, mean annual temperature, spring degree days below 18°C, beginning of frost-free period and distance to roads and trails have relevant ecological importance to the distribution of this alien invasive grass species. While prior research has indicated vegetation community type is important in determining habitat suitability for *B*. *tectorum*, we were not concerned when this variable was dropped based on AICc values; this variable is not static given future potential climates and disturbances such as fire and pine beetle outbreaks in the Park. The logistic probability response indicated 1000 and 1200 spring degree days below 18°C (also known as heating degree days) increases the likelihood of *B*. *tectorum* presence, and this finding is supported by [62], whose study across Western US States including Colorado supported the hypothesis that seed development for this alien grass can be related to cumulative growing degree days at a given geographic location. Furthermore, the current advancement of spring (warmer average temperatures earlier in the year) in the study area will certainly have implications for green-up and reproductive success of *B*. *tectorum*. Since the 1980s, there have been significant snowpack declines along the entire Rocky Mountain range due to warmer spring temperatures [63], which may give this alien an even greater advantage over slower-growing native grasses[64].

Beginning of frost-free period and distance to roads/trails variables contributed equally to the MaxEnt model and should be considered as indicators of *B*. *tectorum* distribution in the Park. Beginning of frost-free period is important when considering release from temperatures that may damage plant tissue. The inclusion of distance to roads in the final model was consistent with prior research of *B*. *tectorum* distribution in the Park [6]; the seeds of *B*. *tectorum* easily attach to vehicle tires and hiking shoes, making roads a key mechanism in its dispersal.

A bioclimatic model at ~ 4 km spatial resolution was developed for the Great Basin ecoregion of the US that indicated summer, annual, and spring precipitation and winter temperatures were the best predictors of *B*. *tectorum* distribution[4]. Coarser resolution SDMs (e.g., greater than 1 km^2^) are useful for large areas such as the Great Basin, but are likely to have greater inherent prediction error in smaller study areas and areas with higher elevational range and topographic heterogeneity such as Rocky Mountain National Park [65]. The *B*. *tectorum* occurrence locations used in this study were spread across a broad elevation gradient, making a finer-scaled SDM important [37]. The 90 m resolution grid cells used in our model combined with climate data that included local weather station data were more likely to capture small areas of climate refugia for *B*. *tectorum* than coarse-scale models that are often used in SDMs.

Our PCA results indicated that some of the ordinal niche space of *B*. *tecotrum* may be conserved in the Park, however a larger portion of this space will shift under future climate conditions. Given the increased within-population genetic variation of *B*. *tectorum* in North American populations and the high phenotypic plasticity of this alien invasive grass [66–68], the future potential shift of its bioclimatic niche space merits constant monitoring for new populations in the Park. One study cited that although *B*. *tectorum* already occurs in Canada, it has the potential to expand this range due to “weedy” genotypes [8]. A more recent study indicated *B*. *tectorum* has the potential to shift phenological development to maximize growth and reproduction [69]. Although quantifying evolutionary adaptations has yet to be realized in SDMs, supplementing these models with PCA may fill the gap in uncertanities associated with niche conservation.

Existing research indicates that climate and soil disturbance are main drivers for successful *B*. *tectorum* invasion [3,70]. Future research modeling *B*. *tectorum* distribution in the Park may incorporate measurements such as soil disturbance that may result in bare ground or loss of competing vegetation, as well as slope and aspect to provide an even stronger case for monitoring particular areas within the Park. *Bromus tectorum* grows in a broad range of soil types; however it is intolerant of shade [1]. A niche model incorporating variables such as slope and light exposure as proxies for shade may provide further inference to *B*. *tectorum* distribution in the Park [36].

The SDM methods outlined in this study provide useful tools for land managers to plan for future potential climates across space and time. An ensemble of six different global circulation models takes into consideration the varying sensitivities to model input among extrapolated climates, thus reducing uncertainty in our projections. This method combined with fine-resolution data from ClimateWNA and the inclusion of annual climate, seasonal climate, and distance to roads/trails produced the most robust estimates of current and future habitat for *B*. *tectorum* in the Park. These maps can improve the efficiency and lower the cost of future surveys. Our methodology can be adopted to generate high resolution species distribution maps under current and future climate scenarios for small study areas and other species, such as the 27 additional invasive species in Rocky Mountain National Park. Land managers can incorporate the maps created from these models into integrated pest management regimes, and further tailor them based on what is already known about an area, keeping in mind that the models must be used in an iterative manner to improve their accuracy. Finally, maps such as these may be displayed to the public to increase awareness of climate change implications in National Parks and beyond.

## Supporting Information

S1 Table Environmental variables (n = 71) evaluated for model inclusion. (DOCX)

S2 Table Akaike’s Information Criterion (AICc) values for a MaxEnt model with differing numbers of variables. (DOCX)
