# Supplementary material for: Using High-Resolution Future Climate Scenarios to Forecast Bromus tectorum Invasion in Rocky Mountain National Park
Source: PLoS One. 2015 Feb 19;10(2):e0117893. doi: 10.1371/journal.pone.0117893 (PMC4335003; doi:10.1371/journal.pone.0117893)
Supplement: S1 Table — (DOCX) [file pone.0117893.s001.docx]

| **Variable description and units of measurement** |
| --- |
| **Annual Variables** |
| Mean annual temperature (°C) |
| Mean warmest month temperature (°C) |
| Mean coldest month temperature (°C) |
| Continentality (°C; difference between MWMT and MCMT) |
| Mean annual precipitation (mm) |
| Mean summer (May to Sept.) precipitation (mm) |
| Annual heat: moisture index (MAT+10)/(MAP/1000) |
| Summer heat:moisture index ((MWMT)/(MSP/1000)) |
| Degree days below 0(°C) |
| Degree days above 5(°C) |
| Degree days below 18(°C) |
| Degree days above 18(°C) |
| Number of frost-free days |
| Frost-free period |
| Julian date on which ffp begins |
| Julian date on which ffp ends |
| Precipitation as snow (mm) between Aug and July |
| Extreme minimum temperature over 30 years |
| Extreme maximum temperature over 30 years |
| Hargreaves reference evaporation |
| Hargreaves climatic moisture deficit |
| **Seasonal Variables** |
| Winter (Dec.(prev. yr) - Feb.) mean temperature (°C) |
| Spring (Mar. - May) mean temperature (°C) |
| Summer (Jun. - Aug.) mean temperature (°C) |
| Autumn (Sep. - Nov.) mean temperature (°C) |
| Winter mean maximum temperature (°C) |
| Spring (Mar. - May) mean maximum temperature (°C) |
| Summer (Jun. - Aug.) mean maximum temperature (°C) |
| Autumn (Sep. - Nov.) mean maximum temperature (°C) |
| Winter (Dec.(prev. yr) - Feb.) mean minimum temperature (°C) |
| Spring (Mar. - May) mean minimum temperature (°C) |
| Summer (Jun. - Aug.) mean minimum temperature (°C) |
| Autumn (Sep. - Nov.) mean minimum temperature (°C) |
| Winter (Dec.(prev. yr) - Feb.) precipitation (mm) |
| Spring (Mar. - May) precipitation (mm) |
| Summer (Jun. - Aug.) precipitation (mm) |
| Autumn (Sep. - Nov.) precipitation (mm) |
| Winter (Dec.(prev. yr) - Feb.) degree days below 0(°C) |
| Spring (Mar. - May) degree days below 0(°C) |
| Summer (Jun. - Aug.) degree days below 0(°C) |
| Autumn (Sep. - Nov.) degree days below 0(°C) |
| Winter (Dec.(prev. yr) - Feb.) degree days above 5(°C) |
| Spring (Mar. - May) degree days above 5(°C) |
| Summer (Jun. - Aug.) degree days above 5(°C) |
| Autumn (Sep. - Nov.) degree days above 5(°C) |
| Winter (Dec.(prev. yr) - Feb.) degree days below 18(°C) |
| Spring (Mar. - May) degree days below 18(°C) |
| Summer (Jun. - Aug.) degree days below 18(°C) |
| Autumn (Sep. - Nov.) degree days below 18(°C) |
| Winter (Dec.(prev. yr) - Feb.) degree days above 18(°C) |
| Spring (Mar. - May) degree days above 18(°C) |
| Summer (Jun. - Aug.) degree days above 18(°C) |
| Autumn (Sep. - Nov.) degree days above 18(°C) |
| Winter (Dec.(prev. yr) - Feb.) number of frost-free days |
| Spring (Mar. - May) number of frost-free days |
| Summer (Jun. - Aug.) number of frost-free days |
| Autumn (Sep. - Nov.) number of frost-free days |
| Winter (Dec.(prev. yr) - Feb.) precipitation as snow |
| Spring (Mar. - May) precipitation as snow |
| Summer (Jun. - Aug.) precipitation as snow |
| Autumn (Sep. - Nov.) precipitation as snow |
| Winter (Dec.(prev. yr) - Feb.) Hargreaves reference evaporation |
| Spring (Mar. - May) Hargreaves reference evaporation |
| Summer (Jun. - Aug.) Hargreaves reference evaporation |
| Autumn (Sep. - Nov.) Hargreaves reference evaporation |
| Winter (Dec.(prev. yr) - Feb.) Hargreaves climatic moisture deficit |
| Spring (Mar. - May) Hargreaves climatic moisture deficit |
| Summer (Jun. - Aug.) Hargreaves climatic moisture deficit |
| Autumn (Sep. - Nov.) Hargreaves climatic moisture deficit |
| **Other Variables** |
| Distance to roads (m) |
| Vegetation Community Type (categorical) |
