# Supplementary material for: Using High-Resolution Future Climate Scenarios to Forecast Bromus tectorum Invasion in Rocky Mountain National Park
Source: PLoS One. 2015 Feb 19;10(2):e0117893. doi: 10.1371/journal.pone.0117893 (PMC4335003; doi:10.1371/journal.pone.0117893)
Supplement: S2 Table — (DOCX) [file pone.0117893.s002.docx]

| **Number of Variables in Maxent Model** | **AICc score** |
| --- | --- |
| 9 | 3975.36 |
| 8 | 3934.77 |
| 7 | 3933.65 |
| 6 | 3918.85 |
| 5 | 3936.18 |
| 4 | 3944.69 |
| 3 | 3984.79 |
| 2 | 4081.40 |
| 1 | 4084.35 |
